# Review: "Neuroethics: defining the issues in theory, practice, and policy" edited by July Illes

**DOI:** 10.1186/1475-925X-5-48

**Published:** 2006-08-02

**Authors:** Monique Frize

**Affiliations:** 1 Department of Systems and Computer Engineering, Carleton University, Ottawa, ON, K1S 5B6, Canada

*Neuroethics *is a collection of essays from thirty-four contributors, presented in three parts: The first part consists of seven essays describing various aspects of neuroscience research and practice, the role of ethics related to this field, selfhood and agency; the debate is focused on whether human decisions, intentions and actions are based on free will or not; the extent with which neuro-scientific knowledge can account for decisions taken by individuals, and whether this is due to determinism (the scientific view) or to autonomous intentional rational agency (the humanistic view). The last article in this section presents ethical dilemmas with regards to autonomy in the face of certain neuro-degenerative diseases like Alzheimer patients.

The second part consists of nine articles on neuro-ethics in practice; this touches on contentious issues such as gene selection, the status of embryos, enhancing brain functionality through technology, research on brain disorders; this section also presents several of the new imaging technologies and the ethical implications of genomic neuro-imaging techniques. New developments of computer-brain interfaces that attempt to interpret brain signals and enable completely paralyzed (locked-in) individuals to communicate or to control devices around them; and developments that attempt to control brain functions as for example the stimulation of the brain to control tremor in patients with Parkinson's disease.

The third part contains five articles that discuss justice, social institutions, and neuroethics. This section deals with the legal, social, and ethical impacts of advances in neuroscience. One essay provides religious responses to neuro-scientific questions and the last article describes how movies and the media portray the human mind.

The writing and tone of the book is quite philosophical and so not an easy read. The book covers a very wide range of topics related to research and practice and ethical implications of the field of neuroscience. The target audience would be neuroscientists, ethicists, practitioners in the fields related to brain function or dysfunction, and educators in these fields. The essays propose a large number of additional references for readers interested to pursue in depth a particular topic. Several interesting case studies are included, which can be useful for the purpose of teaching this subject.

On the down-side, the font used throughout the book is small and even smaller for the case studies, which makes it quite tiring for the eyes. Also, several essays use the exclusive masculine gender, which is annoying in a 2005 publication.

